# Graves’ disease and bone mineral density: a meta-analysis and UK Biobank

**DOI:** 10.3389/fendo.2026.1794055

**Published:** 2026-08-21

**Authors:** Mengchu Wu, Jie Wang, Yunlin Lu, Mengting Yuan, Jiangxun Ji, Junhao Liang, Jiarui Cui, Hongyu Wang, Hongbin Xu, Tianpeng Liu, Yi Shen, Furui Fu, Senjie Shi, Qi Shi, Libing Shen, Dezhi Tang, Chunchun Yuan, Yongjun Wang

**Affiliations:** 1 Longhua Hospital, Shanghai University of Traditional Chinese Medicine, Shanghai, China; 2 Key Laboratory of Theory and Therapy of Muscles and Bones, Ministry of Education, Shanghai, China; 3 Spine Institute, Shanghai Academy of Traditional Chinese Medicine, Shanghai, China; 4 The Affiliated Hospital of Gansu University of Traditional Chinese Medicine, Lanzhou, Gansu, China; 5 Shanghai University of Traditional Chinese Medicine, Shanghai, China; 6 Shanghai Collaborative Innovation Center of Industrial Transformation of Hospital TCM Preparation, Shanghai, China

**Keywords:** bone mineral density, calcium, Graves’ disease, meta-analysis, UK Biobank

## Abstract

**Objective:**

To investigate the association between Graves’ disease (GD) and bone mineral density by integrating a meta-analysis with a large-scale cross-sectional analysis in the UK Biobank.

**Methods:**

We systematically searched PubMed, Web of Science, and Cochrane Library for cohort and cross-sectional studies. The pooled findings were then examined in the UK Biobank using multivariable linear regression models with adjustment for age, BMI, lifestyle factors, vitamin D, calcium, and sex.

**Results:**

The meta-analysis (14 studies, 3,643 participants) showed that GD patients had significantly lower bone mineral density than controls (Mean Difference = −0.09, 95% CI [−0.10, −0.07], *P* < 0.001) and higher serum calcium (Standard Mean Difference = 0.37, 95% CI [0.18, 0.57], *P* = 0.023). However, substantial between-study heterogeneity was observed (I^2^ = 92.1%), indicating considerable variation across studies. Meta-regression and subgroup analyses identified postmenopausal women, older age (>50 years), and lower BMI as key factors amplifying the association. In the UK Biobank (n = 42374), after rigorous adjustment for all covariates, the association was substantially attenuated and remained statistically significant only at the ribs (*P* < 0.05), while other skeletal sites, including the lumbar spine and femoral neck, showed no significant differences.

**Conclusion:**

This dual-evidence approach provides suggestive, rather than definitive, evidence that GD is associated with reduced BMD. The meta-analysis suggested widespread BMD reductions in GD patients. However, due to the limited number of confirmed GD cases, the fully adjusted UK Biobank analysis adopted a broader exposure definition of hyperthyroidism and observed a nominally statistically significant association only at the ribs, with no significant differences detected at other skeletal sites. Given the limited replication of the meta-analysis findings and the high heterogeneity observed in the meta-analysis, we advocate for a risk-stratified approach to BMD screening that prioritizes postmenopausal women, older adults, and individuals with low BMI, rather than routine screening for all GD patients. These findings underscore the need for prospective studies with standardized protocols to clarify the skeletal impact of GD and to guide evidence-based bone health management.

## 1 Introduction

Thyroid hormones are critical regulators of skeletal homeostasis, influencing both bone formation and resorption (1). Consequently, pathological excesses of thyroid hormone, as seen in hyperthyroidism, disrupt this delicate balance, leading to accelerated bone turnover with resorption characteristically outpacing formation (2). This net bone loss significantly increases the risk of osteoporosis and fragility fractures, establishing hyperthyroidism as a major secondary cause of metabolic bone disease.

GD stands as the predominant etiology of hyperthyroidism, accounting for approximately 80% of cases worldwide (3–5). As an organ-specific autoimmune disorder, GD is mediated by stimulating thyrotropin receptor antibodies (TRAb) that drive unregulated thyroid hormone overproduction, culminating in a state of thyrotoxicosis (6, 7). Given its high prevalence, particularly among women, and its fundamental role in inducing a hyperthyroid state, GD represents a clinically relevant scenario where the adverse skeletal effects of thyroid hormone excess are particularly pronounced (8).

Despite the well-established link between hyperthyroidism and bone loss, the specific impact of GD on BMD remains inadequately defined. Existing clinical studies are often limited by small sample sizes, heterogeneous methodologies, and inconsistent findings, which has led to fragmented and often contradictory evidence. This lack of definitive data has created a notable knowledge gap, hindering the development of evidence-based guidelines for BMD screening and management in this large patient population. Consequently, routine bone health monitoring is frequently overlooked in the clinical management of GD, potentially leaving many patients at unmitigated fracture risk.

To address this critical gap, we undertook a comprehensive, dual-evidence investigation. The primary objectives of this study were: 1) to synthesize the existing evidence through a systematic review and meta-analysis to quantify the association between GD and BMD, and 2) to independently validate these findings in a large-scale, prospective cohort using data from the UK Biobank. Through this dual-evidence approach, we aimed to provide more comprehensive evidence to inform clinical practice regarding bone health management in patients with GD.

## 2 Methods

### 2.1 Search strategy

This systematic review and meta-analysis was conducted and reported in accordance with the Preferred Reporting Items for Systematic Reviews and Meta-Analyses (PRISMA) guidelines. The study protocol was registered prospectively in the PROSPERO database (Registration No. CRD42025630482). A comprehensive literature search was performed in the PubMed, Web of Science, and Cochrane Library databases for articles published from database inception to December 2024. The search strategy combined MeSH terms and keywords, including: (“osteoporosis” OR “bone mineral density” OR “BMD” OR “bone density” OR “bone fracture”) AND (“Graves’ disease” OR “Graves’”). No restrictions on language, publication type, or country of origin were applied to maximize sensitivity.

### 2.2 Study selection and data extraction

Following duplicate removal, two reviewers (Mengchu Wu and Jie Wang) independently screened the titles and abstracts of all retrieved records. Full texts of potentially eligible articles were then assessed for final inclusion. Data from all included studies were extracted independently by the same two reviewers using a standardized form. Any discrepancies in screening or data extraction were resolved by consensus or, if necessary, by consultation with a third senior researcher. Studies were eligible for inclusion if they met the following criteria: (1) Population: patients diagnosed with GD (2).Study Design: cross-sectional or cohort studies that included a non-GD control group (3).Outcomes: reported quantitative BMD data (mean and standard deviation) for at least one anatomical site. Exclusion criteria were: (1) reviews, case reports, editorials, or studies lacking original data (2).studies where the intervention group received concurrent osteoporosis therapies in addition to antithyroid drugs (3).populations with other thyroid disorders or significant comorbidities known to affect bone metabolism.

### 2.3 Quality assessment

The methodological quality of all included studies was independently assessed by two reviewers (Mengchu Wu and Jie Wang). Cohort studies were evaluated using the Newcastle-Ottawa Scale (NOS) (9, 10) which assesses participant selection (0–4 points), group comparability (0–2 points), and outcome ascertainment (0–3 points). Cross-sectional studies were appraised using the 11-item Agency for Healthcare Research and Quality (AHRQ) checklist (11). Domain-level scores for each included study are presented in **Supplementary Table 1** and **Supplementary Table 2**. Any disagreements between reviewers were resolved through discussion to reach a consensus, with the involvement of a third senior researcher if needed.

### 2.4 Statistical analysis

All meta-analyses were performed using R software (version 4.3.1). A random-effects model was employed to calculate pooled effect sizes, accounting for anticipated between-study heterogeneity. The magnitude of heterogeneity was assessed using the I^2^ statistic. Pooled effect sizes were expressed as mean difference (MD) for continuous outcomes measured on the same scale, or standardized mean difference (SMD) otherwise, both with 95% confidence intervals (CI). The statistical significance of the overall effect and Cochrane’s Q test for heterogeneity were set at a *P*-value < 0.05. Potential publication bias was evaluated by visual assessment of funnel plot symmetry and formally tested using Egger’s regression test. To explore sources of heterogeneity, subgroup analyses and univariable and multivariable meta-regression analyses were conducted using the restricted maximum likelihood estimation method.

### 2.5 Validation in the UK Biobank cohort

#### 2.5.1 Study population and data source

A cross-sectional analysis was performed using data from the UK Biobank, a large-scale prospective cohort of over 500,000 participants from the United Kingdom. The UK Biobank received ethical approval from the North West Multi-Centre Research Ethics Committee, and all participants provided written informed consent. This analysis was conducted under UK Biobank application number 108866.

#### 2.5.2 Participant selection and exposure definition

Participants were drawn from the full UK Biobank cohort (N = 502,165). We first excluded 456,654 individuals due to missing primary outcome data BMD or incomplete baseline data from the initial selection, yielding a base sample of 45,511 participants with available BMD data. Given the limited number of participants with a confirmed diagnosis of GD alone, we used a broader exposure definition for the primary analysis to ensure sufficient statistical power. Participants were assigned to the hyperthyroidism (including GD) group if they met either of the following criteria: (1) a documented ICD-10 diagnosis code of E05 (thyrotoxicosis) recorded before 2014, or (2) a self-reported non-cancer illness code (Data-Field 2002) of 1225 (thyrotoxicosis) or 1522 (Graves’ disease) with the corresponding date before 2014. This approach is justified because GD accounts for approximately 80% of hyperthyroidism cases, and the skeletal effects are primarily driven by thyroid hormone excess rather than autoimmune-specific mechanisms. Because imaging visits in the UK Biobank occurred between 2014 and 2018, restricting diagnoses to those recorded before 2014 ensures that the exposure preceded BMD measurement in all participants. This approach addresses the temporal ambiguity inherent in a “diagnosis ever” definition and strengthens the temporal interpretability of the analysis.

For the non-exposed control group, we implemented a stringent exclusion protocol. From the screened base sample, we excluded 3649 individuals with any pre-existing medical diagnosis of a thyroid disorder, self-reported thyroid problems or dysfunction, or a history of thyroid-related medication use or surgery. Following these exclusions, 42374 participants were enrolled in the final control group. This process yielded a final analytic cohort of 42374 participants (41862 controls and 512 hyperthyroidism cases).

#### 2.5.3 Outcomes and statistical analysis

The primary outcomes were BMD measured by DXA at the following skeletal sites: L1–L4 spine, femoral neck, femoral shaft, total hip, trochanter, Ward’s triangle, as well as arms, head, legs, pelvis, ribs, spine, total body, and trunk. To quantify the association between hyperthyroidism and BMD, multivariable linear regression models were fitted for each BMD outcome, with full adjustment for age, sex, BMI, smoking status, alcohol intake, physical activity (IPAQ), serum levels of vitamin D and calcium. Subgroup analyses were performed to explore heterogeneity and identify high-risk populations, including treatment subgroups (untreated hyperthyroidism, medication only, surgery only, and medication plus surgery) and sex subgroups (males, postmenopausal females, and premenopausal females).

To handle missing covariate data, multiple imputation by chained equations (MICE) was applied consistently across the entire analysis cohort, stratified by hyperthyroidism status to preserve group-specific variance structures. Under the standard missing at random (MAR) assumption, we generated M = 20 imputed datasets. This choice follows current methodological recommendations, which suggest that 20 or more imputations are needed to obtain stable estimates of standard errors, confidence intervals, and *P*-values, beyond the classic 3–5 imputation rule that primarily ensures efficiency for point estimates. The imputation models included all 19 BMD outcome variables, the primary exposure (hyperthyroidism status), and all multivariable adjustment covariates (age, sex, BMI, vitamin D supplementation, calcium supplementation, smoking status, alcohol consumption, and physical activity via IPAQ). The missing data proportions across the adjusted covariates ranged from 0.67% to 18.75% (**Supplementary Table 3**). Convergence of the MICE algorithm was verified by examining the iteration history, which showed stable estimates across successive iterations, indicating proper convergence.

Menopausal status was not included as a covariate in the whole-cohort imputation or primary models, as it is biologically applicable only to female participants and would be structurally missing for all males. Instead, it was retained exclusively in female subgroup analyses, where it was dynamically imputed using age as the primary auxiliary variable. Linear regression analyses were performed in parallel on each of the 20 imputed datasets, and the resulting point estimates and standard errors were pooled using Rubin’s rules to generate final multivariable-adjusted β coefficients with 95% confidence intervals. All analyses were performed using R software (version 4.3.1), and a two-sided *P* < 0.05 was considered statistically significant.

## 3 Results

### 3.1 Study selection and characteristics

**Figure 1:**
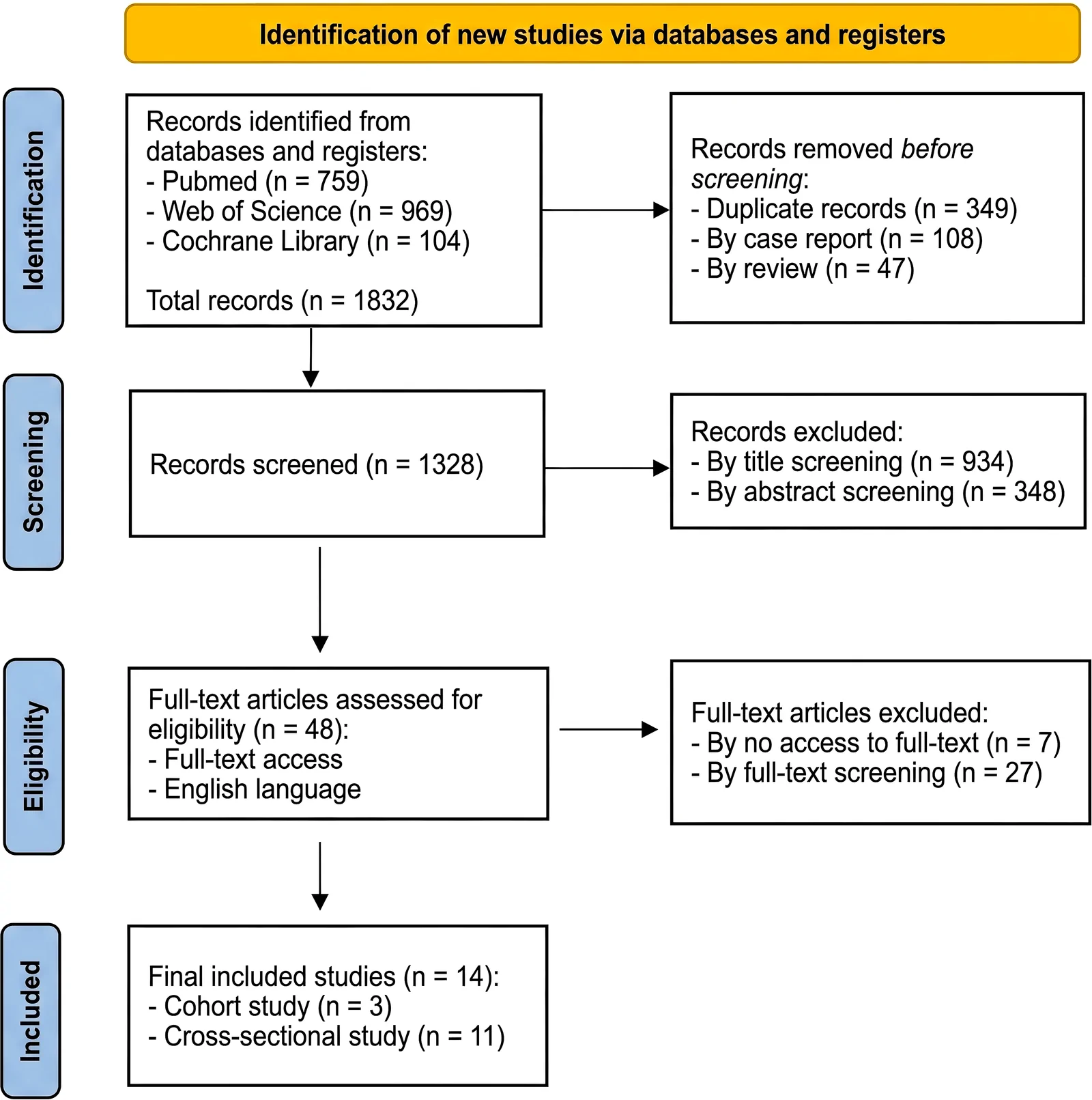
PRISMA flow diagram of study selection.

The systematic literature search initially identified 1,832 records from PubMed (n = 759), Web of Science (n = 969), and Cochrane Library (n = 104). After removing 349 duplicates, 1,328 records were screened by title and abstract, during which 155 records were excluded (108 case reports and 47 reviews). The remaining 48 full-text articles were assessed for eligibility, of which 7 were excluded due to no access to full-text and 27 were excluded after full-text screening. Ultimately, 14 observational studies met the inclusion criteria and were included in the final meta-analysis, comprising 11 cross-sectional studies and 3 cohort studies. The detailed selection process is illustrated in the PRISMA flow diagram (**Figure 1**). The 14 included studies involved a total of 3,643 participants and consisted of two distinct designs. **Table 1** summarizes the characteristics of the 11 cross-sectional studies (12–22), which compared GD patients to healthy controls. Across these studies, which were predominantly conducted in Asian populations, GD patient groups were largely female and consistently exhibited a lower mean BMI compared to control groups. **Table 2** details the characteristics of the 3 longitudinal cohort studies (23–25). These studies focused on tracking BMD changes in GD patients before and after antithyroid drug therapy, providing specific data on treatment regimens and duration. The methodological quality of all included studies was generally high, as assessed by the NOS for cohort studies and the AHRQ checklist for cross-sectional studies.

**Table 1:** Baseline characteristics of Graves’ disease cohort versus healthy controls.

No	First author	Country	Type	N	Age	Sex (M/F)	BMI	N (c)	Age (c)	Sex M/F (c)	BMI (c)	Lumbar BMD (GD/c)	AHRQ/NOS
		Year											
1	Boro (12)	India 2023	cross- sectional	50	31.9± 10.9	19/31	20.8 ± 4	50	31.2± 4.9	19 /31	25.7 ± 4.4	0.84 ± 0.146/0.96 ± 0.096	8
2	Lee,H.S (13)	Korea 2021	cross- sectional	41	12.1± 2.2	9/32	–	172	11.7± 1.8	41 /131	–	0.87 ± 0.186/0.88 ± 0.162	7
3	Siderova (14)	Bulgaria 2018	cross- sectional	47	55.6± 12.8	11/36	25.3 ± 3.23	40	53.2± 7.1	10/30	26.1 ± 3.5	0.99 ± 0.15/1.20 ± 0.08	8
4	Kuzma (15)	Slovakia 2018	cross- sectional	21	35.2± 2.2	0/21	23.1 ± 0.9	23	33.1± 2.1	0/23	22.2 ± 0.8	1.02 ± 0.03/1.05 ± 0.03	8
5	Biswas (16)	India 2015	cross- sectional	31	37± 10.4	15/16	18.4 ± 2.4	30	38.5± 7.5	14/16	19.8 ± 1.8	1.01 ± 0.10/1.13 ± 0.16	7
6	Amashukeli (17)	Georgia 2013	cross- sectional	20	55.8± 4.7	0/20	26.6 ± 5.5	30	55.3± 4.8	0/30	29.6 ± 2.7	0.83 ± 0.153/0.99 ± 0.115	7
7	Majima-2 (18)	Japan 2006	cross- sectional	56	43.6± 10	56/0	22.9 ± 2.1	34	43.8± 10.5	34/0	22.9 ± 2	0.96 ± 0.095/1.02 ± 0.078	7
8	Altun (19)	Turkey 2003	cross- sectional	21	36± 2	0/21	25 ± 0.8	10	35± 3	0/10	26± 0.9	–	7
9	Leary(I) (20)	Ireland 2001	cross- sectional	28	53.8± 11.8	0/28	–	100	53.2± 8.2	0/100	–	–	6
9	Leary(II) (20)	Ireland 2001	cross- sectional	16	56.7± 11.9	0/18	–	33	49.4± 6.1	0/33	–	–	6
10	Lee,M.S (I) (21)	Korea 1990	cross- sectional	6	–	6/0	–	6	–	6/0	–	0.99 ± 0.18/0.99 ± 0.115	7
10	Lee,M.S (II) (21)	Korea 1990	cross- sectional	28	–	0/28	–	28	–	0/28	–	0.90 ± 0.182/1.00 ± 0.082	7
10	Lee,M.S (III) (21)	Korea 1990	cross- sectional	22	<50	0/22	–	22	<50	0/22	–	0.98 ± 0.126/1.04 ± 0.031	7
10	Lee,M.S (IV) (21)	Korea 1990	cross- sectional	6	>50	0/6	–	6	>50	0/6	–	0.68 ± 0.107/0.88 ± 0.045	7
11	Ercolano(I) (22)	Argentina 2013	cross- sectional	27	56.6± 5.5	0/27	26.1 ± 4.5	16	54.8± 7.4	0/16	28.6 ± 4.7	1 ± 0.17/1.06 ± 0.13	7
11	Ercolano(II) (22)	Argentina 2013	cross- sectional	30	38.8± 9.7	0/30	25.2 ± 3.8	36	36.3± 10.6	0/36	23.2 ± 4.2	1.19 ± 0.15/1.17 ± 0.12	7
12	Jyotsna (24)	India 2012	cohort	80	36.3± 11.1	18/62	20.4 ± 3.48	80	36.4± 10.4	–	23.6 ± 4.0	0.86 ± 0.141/0.91 ± 0.2	8
13	Majima-1 (I) (25)	Japan 2006	cohort	14	43.6± 11	14/0	22.8 ± 1.9	34	43.8± 10.5	34/0	22.9 ± 2	0.94 ± 0.102/1.02 ± 0.078	8
13	Majima-1 (II) (25)	Japan 2006	cohort	13	45.4± 13.1	13/0	22.3 ± 1.8	34	43.8± 10.5	34/0	22.9 ± 2	0.95 ± 0.124/1.02 ± 0.078	8
14	Zhang (23)	China 2022	cohort	758	41.2± 12.2	237/521	20.9 ± 2.7	1164	41.1± 10.4	366/798	23.6 ± 3.3	1.02 ± 0.136/1.13 ± 0.268	9

Data are presented as mean ± SD. Dashes (-) indicate unavailable data; “(c)” denotes control group. Studies with multiple datasets are labeled as follows: Leary(I/II), Lee,M.S(I-IV), Ercolano(I/II), Majima-1(I/II), and Majima-2.

**Table 2:** Baseline characteristics of GD patients before and after treatment.

No	First author	Year	Country	Type	N	Age	Sex (M/F)	BMI	Drug	Dose	Treat time (month)	NOS
1	Zhang (23)	2022	China	cohort	287	40.0± 12.6	85/202	23.1± 0.9	–	–	24	9
2	Jyotsna (24)	2012	India	cohort	80	36.3± 11.1	18/62	22.9± 2.1	MMI	30-60mg/day	12/24	8
3	Majima-1 (II) (25)	2006	Japan	cohort	13	45.4± 13.1	13/0	22.3± 1.8	MMI	30mg/day	6/12	8

Data presented as mean ± SD unless specified. Dashes (–) indicate unavailable data.

### 3.2 Meta-analysis findings

#### 3.2.1 BMD analysis

**Figure 2:**
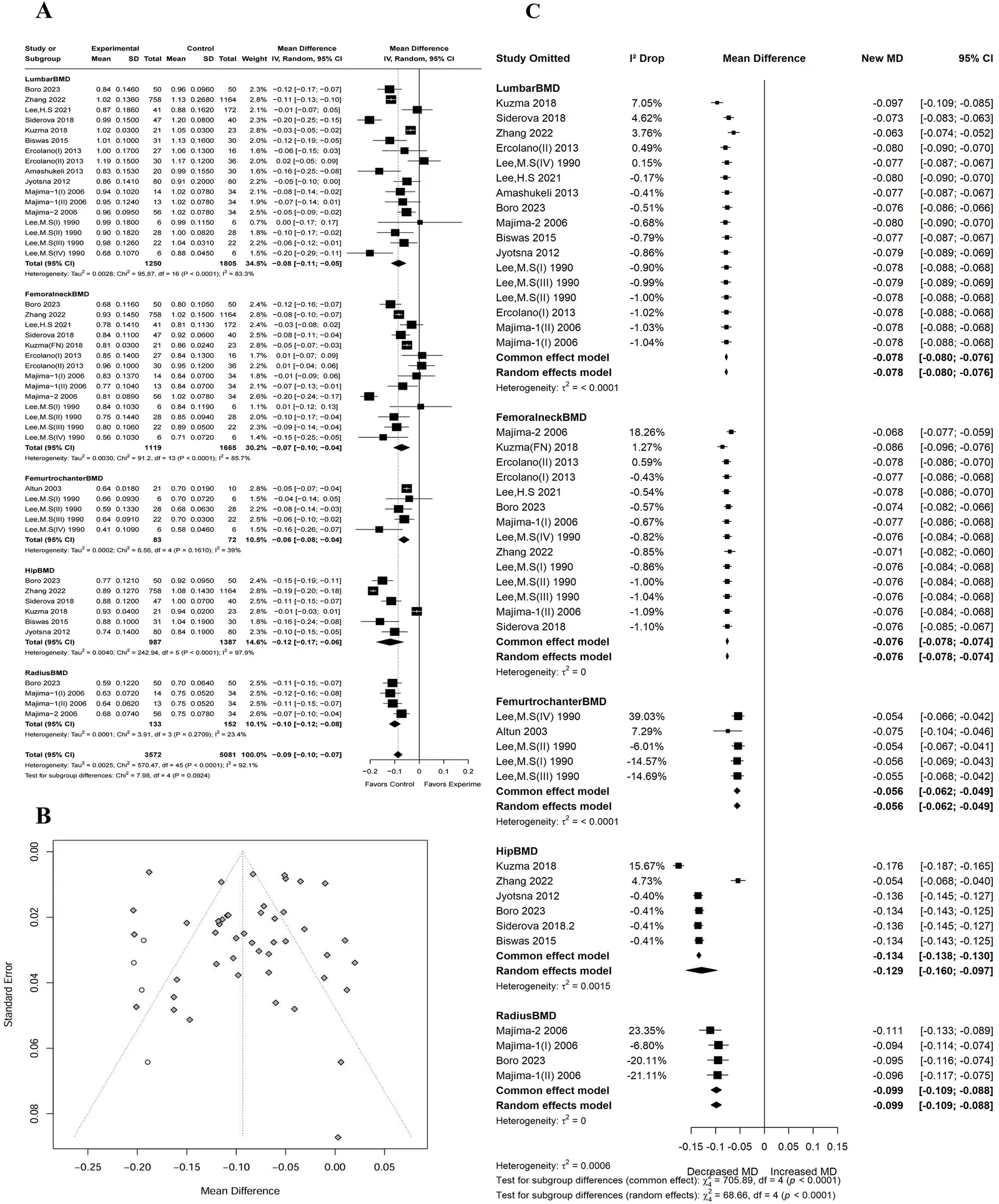
Meta-analysis of the association between Graves’ disease and BMD. **(A)** Forest plot of the MD in BMD between patients with Graves’ disease and healthy controls, stratified by anatomical site. **(B)** Funnel plot used to assess publication bias in the BMD meta-analysis. **(C)** Leave-one-out sensitivity analysis showing that no single study drives the overall effect, and the observed heterogeneity is likely attributable to differences in study populations and measurement protocols.

Subgroup analyses by anatomical sites revealed significant differences in BMD between GD patients and controls (**Figure 2A**). The lumbar spine (12–18, 21–25) showed a MD of -0.08 (I^2^ = 83.3%, 95% CI [-0.11, -0.05], *P* < 0.001), while the femoral neck (12–15, 18, 21–23, 25) exhibited an MD of -0.07 (I^2^ = 85.7%, 95% CI [-0.10, -0.04], *P* < 0.001).The total hip (12, 14–16, 23, 24) demonstrated the most pronounced reduction (MD = -0.12, I^2^ = 97.9%, 95% CI [-0.17, -0.06], *P* < 0.001). The pooled analysis showed a significant overall BMD reduction in GD patients (MD = -0.09, I^2^ = 92.1%, 95% CI [-0.10, -0.07], *P* < 0.001), with substantial heterogeneity (I^2^ > 80%) indicating considerable variation between studies. Subgroup analysis suggested significant differences across anatomical sites (*P* = 0.0189). Funnel plot analysis (**Figure 2B**) and Egger’s test (*P* = 0.641) found no evidence of publication bias. However, it should be noted that Egger’s test has limited statistical power when the number of included studies is small (typically < 10). For subgroup analyses with fewer studies (e.g., femoral trochanter, k = 5; radius, k = 4), the absence of significant publication bias should be interpreted with caution.

**Figure 3:**
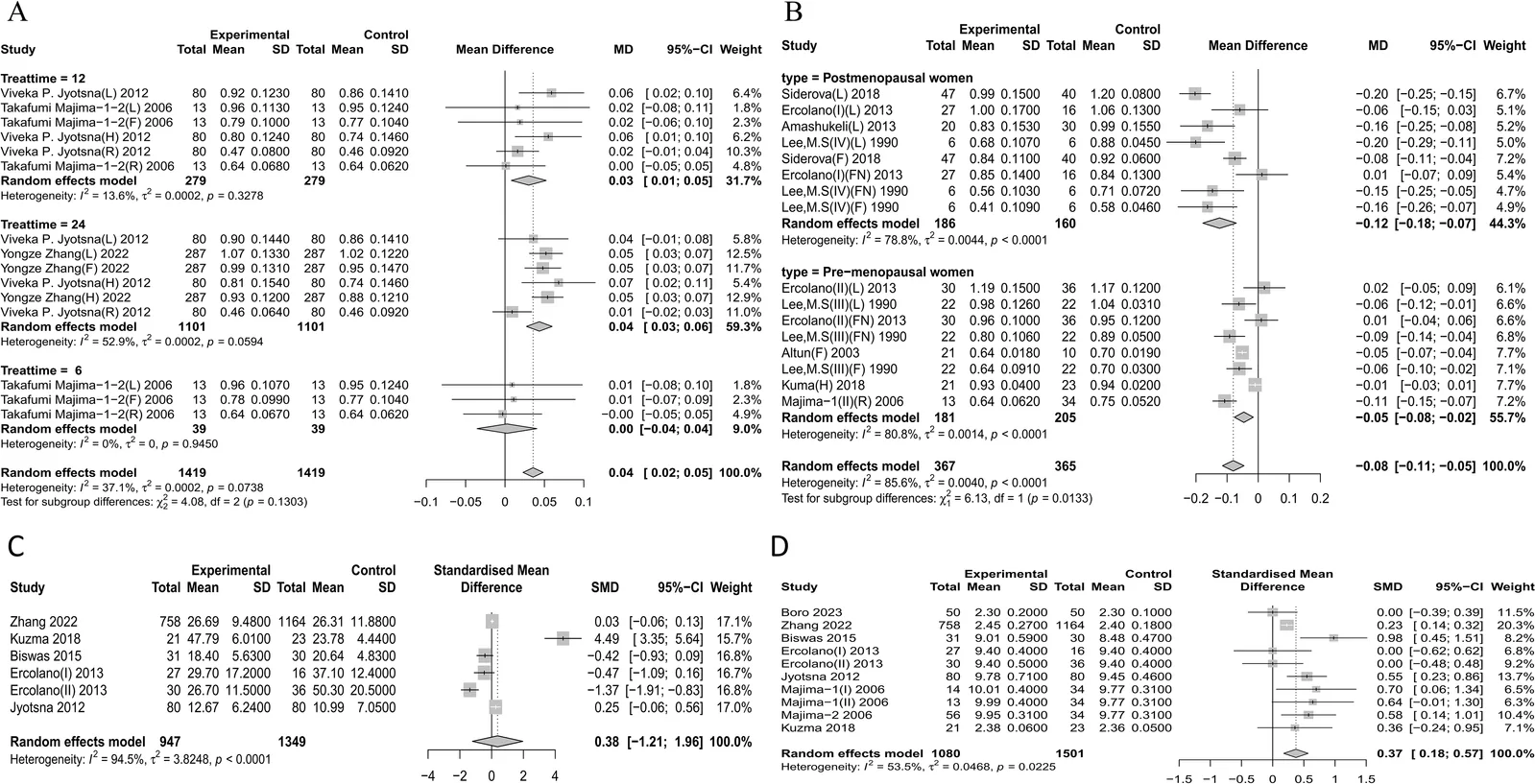
Meta-analyses of biochemical markers, longitudinal BMD changes, and menopausal subgroup analysis. **(A)** Longitudinal BMD changes after antithyroid therapy (6, 12, 24 months). **(B)** BMD differences in female GD patients vs. controls, stratified by menopausal status. Abbreviations: FN, femoral neck; F, femur trochanter; H, hip; L, lumbar spine; R, radius. **(C)** Serum vitamin D levels (GD vs. controls). **(D)** Serum calcium levels (GD vs. controls).

To explore the sources of this heterogeneity and assess the robustness of the pooled estimates, we performed a leave-one-out sensitivity analysis (**Figure 2C**). This analysis demonstrated that no single study drove the overall effect, as the pooled mean difference remained consistently significant after sequentially omitting each study. However, given the substantial residual heterogeneity, the pooled estimate should be interpreted as a summary of the available evidence rather than a precise or universal effect size. Longitudinal analysis of BMD changes following antithyroid therapy showed a trend toward improvement, but this did not reach statistical significance (MD = 0.04, I^2^ = 37.1%, 95% CI [0.02, 0.05], *P* = 0.074) (**Figure 3A**). Subgroup analysis by menopausal status revealed distinct patterns: postmenopausal women showed greater BMD reduction (14, 17, 21, 22) (MD = -0.12, I^2^ = 78.8%, 95% CI [-0.18, -0.07], *P* < 0.00) compared to premenopausal women (15, 19, 21, 22, 25) (MD = -0.05, I^2^ = 80.8%, 95% CI [-0.08, -0.02], *P* < 0.001). The difference between these groups was statistically significant (*P* = 0.0133) (**Figure 3B**). These findings suggest that postmenopausal GD patients maybe at higher risk for BMD reduction, particularly at the lumbar spine.

#### 3.2.2 Vitamin D and serum calcium analysis

The analysis of vitamin D levels included six studies (15, 16, 22–24) and revealed no statistically significant differences between GD patients and controls (SMD = 0.38, 95% CI [-1.21, 1.96], *P* > 0.05). However, substantial heterogeneity was observed across studies (I^2^ = 94.5%), with SMDs ranging from -1.37 to 4.49 (**Figure 3C**). In contrast, the meta-analysis of serum calcium levels (12, 15, 16, 18, 22–25) involving 10 studies, revealed significantly higher levels in GD patients compared to healthy controls (SMD = 0.37, 95% CI [0.18, 0.57], *P* = 0.023), with moderate heterogeneity (I^2^ = 53.5%) (**Figure 3D**).

#### 3.2.3 Meta-regression analysis

To explore potential sources of heterogeneity, we performed univariable meta-regression analyses incorporating prespecified covariates, including BMD measurement site, geographical region, BMI, age, and sex (**Table 3**). Variables with the strongest signals in univariable analyses (BMI, age, and region) were then entered into a multivariable meta-regression model. The final multivariable model (32 study arms, residual I^2^ = 87.06%) explained 38.98% of the between-study variance (R2 = 38.98%, *P* = 0.0016). In this model, age > 50 years was significantly associated with lower BMD (β = −0.1149, *P* = 0.0025), while BMI > 24 kg/m² showed a protective effect (β = 0.0726, *P* = 0.037) (**Table 4**). Substantial residual heterogeneity remained (I^2^ = 87.06%), suggesting that unmeasured factors—such as treatment duration, disease severity, genetic background, or nutritional status—may also contribute to between-study variation.

**Table 3:** Univariate meta-regression analysis of heterogeneity sources.

Variable	R² (%)	*P*-val	I² (%)	k
Age	12.57	0.0595	91.01	37
Sex	0	0.6095	89.58	46
BMD	0	0.5118	87.67	46
BMI	11.45	0.0739	91.1	32
Region	8.32	0.0639	88.46	46

**Table 4:** Multivariate meta-regression analysis of heterogeneity determinants.

Variable	*P*-val	β
BMI > 24	0.037	0.0726
BMI 22-24	0.2018	0.0342
Age > 50	0.0025	-0.1149
Age 40-49	0.7082	-0.0102
Non-Asian regions	0.1189	0.0527

The multivariate meta-regression analysis revealed the following overall model results (I² = 87.06%, R² = 38.98%, *P* = 0.0016).

### 3.3 UKB validation study

#### 3.3.1 Participant characteristics

**Figure 4:**
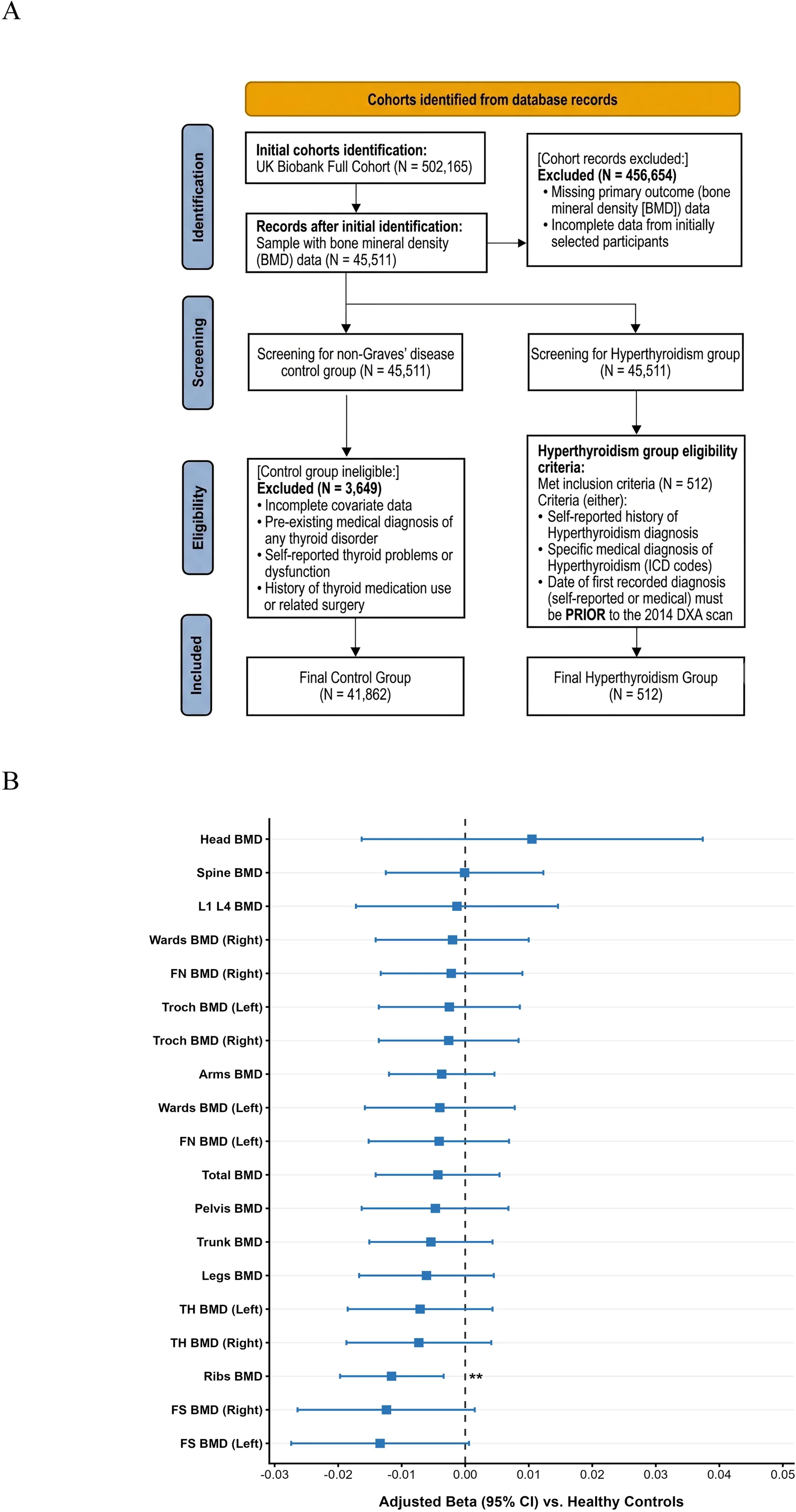
**(A)** Flowchart of participant selection from the UK Biobank cohort. **(B)** Forest plot comparing BMD levels at various anatomical sites between the hyperthyroidism cohort and the non-hyperthyroidism control group. Results are presented as adjusted beta coefficients with 95% CI. **P < 0.05.

The final cross-sectional analysis (**Figure 4A**) included 42374 participants, comprising 41862 individuals in the control group and 512 patients with hyperthyroidism. Consistent with the established epidemiological profile of the condition, a significant sex disparity was observed; females constituted a substantially larger proportion of the hyperthyroidism cohort compared to the control group (78.7% vs. 49.3%, *P* < 0.001). Patients with hyperthyroidism were marginally older (65 ± 8 vs. 64 ± 8 years, *P* = 0.070), and BMI did not differ significantly between the two groups (26.7 ± 4.8 vs. 26.5 ± 4.4 kg/m², *P* = 0.741). Furthermore, the baseline assessment revealed significant differences in lifestyle behaviors and supplement intake. The hyperthyroidism group exhibited distinct patterns of alcohol intake frequency (*P* < 0.001) and reported a higher prevalence of low physical activity levels (16% vs. 12%, *P* = 0.012) compared to controls. Additionally, patients with hyperthyroidism reported significantly higher rates of both vitamin D (5.3% vs. 1.9%, *P* < 0.001) and calcium supplementation (4.0% vs. 0.8%, *P* < 0.001). These baseline characteristics highlight important population stratification factors that will be accounted for as covariates in subsequent statistical models.

#### 3.3.2 Association between hyperthyroidism and BMD

In multivariable linear regression models fully adjusted for all covariates (**Figure 4B**), hyperthyroidism was associated with significantly reduced BMD at specific skeletal sites, including the ribs (all P < 0.05). In contrast, no statistically significant differences in BMD were observed at the other skeletal sites, including the head, spine (including L1-L4), appendicular skeleton (arms and legs), proximal femur (including femoral neck, trochanter, and Wards triangle), and total body (all *P* > 0.05). In sex- and menopause-stratified analyses (**Supplementary Table 4**), menopausal status was dynamically imputed exclusively within the female subgroup. Significant BMD reductions were observed at the ribs in premenopausal females, and at the femoral shaft in premenopausal females (all *P* < 0.05). A post-hoc change-in-estimate analysis further confirmed that sex accounted for the majority of the attenuation in the hyperthyroidism-BMD association, while adjustment for other covariates resulted in minimal additional change (**Supplementary Table X**).

#### 3.3.3 Impact of clinical interventions on BMD

To further explore the potential influence of clinical management on skeletal health, the hyperthyroidism cohort was stratified into four treatment subgroups: untreated, drug only, surgery only, and combined therapy (**Supplementary Figure 1**). In these exploratory analyses, adjusted for available covariates, the untreated subgroup showed significant BMD reductions at the ribs and femoral shaft compared with controls (all *P* < 0.05). No significant differences were observed at other skeletal sites, nor in any of the treated subgroups (drug only, surgery only, or combined therapy; all *P* ≥ 0.05). Given the exploratory nature of these analyses and the possibility of residual confounding due to unmeasured factors, these findings should be interpreted cautiously and are not intended to support causal inferences regarding treatment effects.

## 4 Discussion

**Figure 5:**
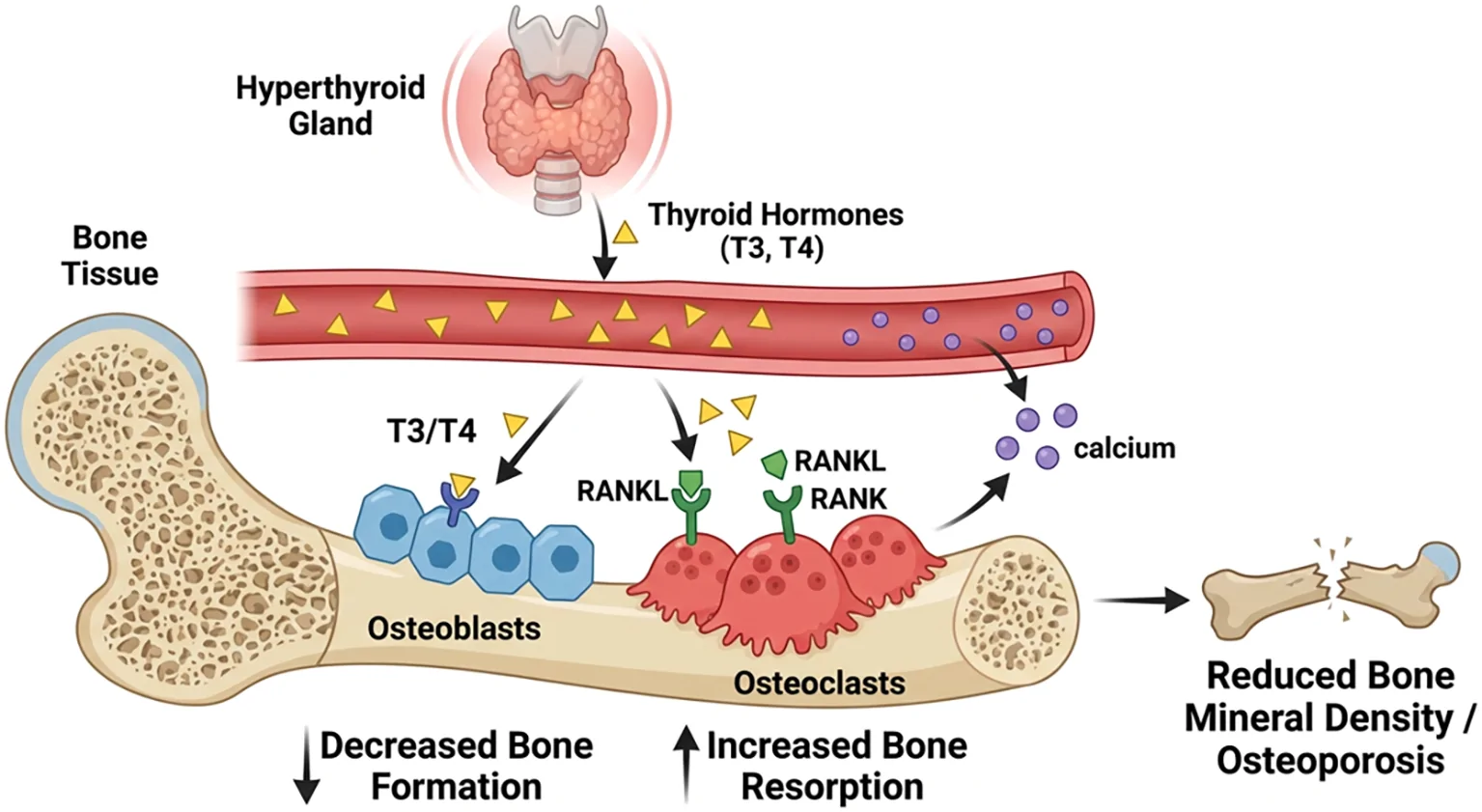
Schematic illustration of the proposed mechanism by which hyperthyroidism contributes to reduced bone mineral density. Excess thyroid hormones (T3 and T4) suppress osteoblast-mediated bone formation and promote RANKL/RANK-mediated osteoclast activation and bone resorption, increasing calcium release from bone and ultimately contributing to reduced BMD and the development of osteoporosis.

Our study presents a complex picture regarding the association between GD and BMD. The meta-analysis suggested widespread BMD reductions across multiple skeletal sites, consistent with the known biological effects of thyroid hormone excess on bone metabolism. However, in the UK Biobank, after rigorous adjustment for all covariates, a statistically significant reduction in BMD was observed only at the ribs, with no significant associations detected at other skeletal sites. This discrepancy between the meta-analysis and UK Biobank findings suggests that confounding factors, particularly sex, may have substantially contributed to the positive results reported in previous literature. This biological framework is supported by our meta-analysis findings, as detailed below. Mechanistic basis lies in the disruption of skeletal homeostasis driven by thyroid hormone excess. Hyperthyroidism accelerates the bone remodeling cycle, disproportionately enhancing osteoclast-mediated resorption while compensatory formation cannot keep pace (26) (**Figure 5**). This process is compounded by hyperthyroidism-induced mineral dysregulation: excessive resorption releases calcium into circulation, causing hypercalcemia that suppresses PTH secretion (27, 28). The suppression of PTH further exacerbates the problem by reducing intestinal calcium absorption and increasing renal calcium excretion, culminating in a sustained negative calcium balance that depletes skeletal mineral reserves (29–31) (**Supplementary Figure 2**).

Our findings on serum biomarkers align with this pathophysiological model. The observed higher serum calcium levels in GD patients are consistent with excessive bone resorption. Conversely, our analysis revealed no significant difference in serum vitamin D levels between GD patients and controls. However, this finding should be interpreted with substantial caution due to the very high heterogeneity observed across the included studies (I^2^ = 94.5%). Given the considerable variation in effect sizes and the observational nature of the data, a potential contributory role of vitamin D deficiency in specific clinical contexts cannot be entirely ruled out (**Supplementary Figure 3**).Our longitudinal analysis explored whether reversing hyperthyroidism mitigates bone loss. While restoring euthyroidism is fundamental, BMD recovery may be a slow process. We observed a numerical trend toward BMD improvement following antithyroid therapy over 24 months; however, this change did not reach statistical significance (MD = 0.04, *P* = 0.074). This may be attributable to the limited number of longitudinal studies (n = 3) or short follow-up durations (6–24 months). Thus, evidence for BMD recovery after treatment should be interpreted cautiously, underscoring the importance of early intervention before significant bone mass is lost (**Supplementary Figure 4**). It is also important to acknowledge the apparent discrepancy between the meta-analysis, which showed widespread BMD reductions at multiple skeletal sites, and the multivariable-adjusted UK Biobank analysis, where the independent effect of GD remained significant only at the ribs and femoral shaft. This divergence is likely explained by differences in confounding control and statistical power. The meta-analysis primarily included studies that did not adequately adjust for sex, a strong confounder in the relationship between GD and BMD. In contrast, the UK Biobank analysis applied rigorous adjustment for sex and other covariates, thereby isolating the independent effect of GD. Additionally, the relatively small number of GD cases in the UK Biobank (n = 568) may have limited statistical power to detect significant differences at other skeletal sites.

Beyond the primary association, our study identified key patient subgroups at heightened risk, where the effects of GD are amplified. Postmenopausal women with GD exhibited a significantly more pronounced reduction in BMD. This may represent a “double-hit” effect, where the accelerated bone resorption from hyperthyroidism is compounded by the loss of estrogen’s protective, anti-resorptive effects. Existing research indicates that the abrupt estrogen decline during menopause constitutes a primary driver of BMD reduction (30). Estrogen exerts its protective effects by inhibiting osteoclastogenesis and suppressing bone resorption through osteoclast activity blockade. The postmenopausal state exacerbates this process through estrogen deficiency, which further enhances osteoclast activity and accelerates BMD deterioration (32–34).

Our meta-regression confirmed that older age (> 50 years) and lower BMI are strong, independent predictors of lower BMD in the GD population. Osteoporosis is an age-related condition characterized by progressive BMD decline that accelerates with aging (35). Multiple studies consistently demonstrate that higher BMI correlates with attenuated BMD loss (36–38). The protective effect of higher BMI is likely twofold: increased mechanical loading stimulating bone formation (39), and greater adipose tissue-derived estrogen production, which becomes a crucial source of anti-resorptive signaling after menopause (40, 41) (**Supplementary Figure 5**).

Our findings have several practical implications for the management of GD patients. GD is associated with a significant reduction in bone mineral density, as confirmed by meta-analysis. However, sex is a strong confounder. After rigorous multivariable adjustment in the UK Biobank, the independent effect of GD persisted only at the ribs and femoral shaft. High-risk subgroups include postmenopausal women, adults over 50 years of age, and individuals with low body mass index, and these populations should undergo targeted bone mineral density screening. In conclusion, this dual-evidence approach provides suggestive evidence-that GD is associated with reduced BMD, although substantial between-study heterogeneity and the partial replication in the UK Biobank temper the certainty of this conclusion. The meta-analysis suggested widespread BMD reductions, whereas the fully adjusted UK Biobank analysis identified significant associations only at selected skeletal sites, indicating that the independent effect of GD may be more modest and site-specific than previously assumed. The underlying mechanism is primarily driven by thyroid hormone-induced acceleration of bone resorption, as supported by the observed higher serum calcium levels in GD patients. Meta-regression and subgroup analyses identified postmenopausal women, older adults (>50 years), and individuals with low BMI as high-risk subgroups who are disproportionately affected. Based on these findings, we advocate for a risk-stratified approach to BMD screening that prioritizes these vulnerable populations, rather than routine screening for all GD patients. Future prospective studies with standardized protocols and detailed treatment histories are needed to further clarify the skeletal impact of GD and to inform evidence-based bone health management in this population.

## 5 Limitations

This study has several limitations. First, we could not perform meta-analyses on key bone turnover markers (PTH, P1NP, CTX) because most primary studies reported these non-normally distributed data as medians and IQRs, precluding quantitative synthesis. Additionally, the UK Biobank did not collect data on these bone turnover markers, nor did it provide information on thyroid hormone levels or TRAb titers at the time of BMD measurement; therefore, we were unable to determine whether GD was in an active or remitted state, which limited our ability to assess the impact of disease activity on bone loss.

Second, substantial heterogeneity (I^2^ > 80%) was observed in our primary meta-analyses, likely reflecting variations in DXA protocols, unmeasured confounders (e.g., smoking, physical activity, calcium intake), and disease-specific factors (e.g., hyperthyroidism severity, treatment regimens). The lack of individual patient data precluded a more granular exploration.

Third, the UK Biobank analysis was constrained by a relatively small number of hyperthyroidism cases (n = 512) and an even smaller number of confirmed GD cases, leading to a significant case-control imbalance and limited statistical power to detect smaller effect sizes. Additionally, the broad exposure definition (ICD-10 E05, encompassing all thyrotoxicosis) may have introduced exposure misclassification, potentially biasing estimates toward the null. Furthermore, the exposure definition used in the UK Biobank (hyperthyroidism including GD) was not identical to that in the meta-analysis (GD alone). This discrepancy limits the direct comparability of the two analyses and should be considered when interpreting the partial replication of the meta-analysis findings. Additionally, the UK Biobank lacked detailed data on the exact timing of treatment initiation relative to BMD measurement, as well as consistently recorded treatment modalities (antithyroid medication, thyroidectomy, or combined therapy), precluding a reliable assessment of treatment-specific effects. Future large-scale studies with more precise phenotyping are needed to confirm these findings. Fourth, our analysis did not account for autoimmune comorbidities commonly associated with GD, such as type 1 diabetes mellitus, celiac disease, and autoimmune gastritis, which are independently linked to reduced bone mineral density. The absence of such data in the primary studies and the UK Biobank limited our ability to adjust for these potential confounders. Fifth, the discrepancy between the meta-analysis, which showed widespread BMD reductions, and the fully adjusted UK Biobank analysis, in which significant reductions persisted only at the ribs and femoral shaft, may reflect multiple sources of heterogeneity, including population differences between Asian and European ancestry and sex-related heterogeneity. The association was substantially attenuated after full covariate adjustment in the UK Biobank, whereas most primary studies in the meta-analysis did not adequately adjust for sex, potentially overestimating the independent effect of GD on BMD. Additionally, potential interactions between menopausal status and sex may further contribute to this discrepancy, collectively limiting the external validity of our findings. Finally, publication bias cannot be entirely ruled out for subgroup analyses with fewer than 10 studies (e.g., femoral trochanter, radius).

## 6 Conclusions

In conclusion, this dual-evidence approach provides suggestive evidence that GD is associated with reduced BMD, although substantial between-study heterogeneity and the partial replication in the UK Biobank temper the certainty of this conclusion. The meta-analysis suggested widespread BMD reductions, whereas the fully adjusted UK Biobank analysis identified significant associations only at selected skeletal sites, indicating that the independent effect of GD may be more modest and site-specific than previously assumed. The underlying mechanism is primarily driven by thyroid hormone-induced acceleration of bone resorption, as supported by the observed higher serum calcium levels in GD patients.

Meta-regression and subgroup analyses identified postmenopausal women, older adults (>50 years), and individuals with low BMI as high-risk subgroups who are disproportionately affected. Based on these findings, we advocate for a risk-stratified approach to BMD screening that prioritizes these vulnerable populations, rather than routine screening for all GD patients. Future prospective studies with standardized protocols and detailed treatment histories are needed to further clarify the skeletal impact of GD and to inform evidence-based bone health management in this population.

## Data Availability

The UK Biobank data analyzed in this study are available to bona fide researchers upon application to the UK Biobank (https://www.ukbiobank.ac.uk), subject to approval and compliance with the applicable data-access policies. This study used UK Biobank data under application number 108866. Data supporting the meta-analysis are included in the article and **Supplementary Material**.

## Supplementary material

Supplementary data file 1 Cohort study data used in the longitudinal meta-analysis. This file contains the extracted study-level data for changes in bone mineral density following antithyroid treatment, including the first author, publication year, anatomical measurement site, sample sizes, mean BMD values, standard deviations, and follow-up duration at 6, 12, or 24 months.

Supplementary data file 2 Cross-sectional study data used in the meta-analysis and subgroup analyses. This file contains the extracted study-level data for bone mineral density, serum 25-hydroxyvitamin D, serum calcium, body mass index, and menopausal status. It includes sample sizes, means, standard deviations, and relevant subgroup variables such as sex, age category, geographical region, BMI category, and menopausal status.
