# Supplementary material for: Graves’ disease and bone mineral density: a meta-analysis and UK Biobank
Source: Front Endocrinol (Lausanne). 2026 Aug 21;17:1794055. doi: 10.3389/fendo.2026.1794055 (PMC13541481; doi:10.3389/fendo.2026.1794055)
Supplement: Supplementary file 1 [file SupplementaryFile1.docx]

Supplementary Material

# 1 Supplementary Data

Supplementary Material should be uploaded separately on submission. Please include any supplementary data, figures and/or tables.

Supplementary material is not typeset so please ensure that all information is clearly presented, the appropriate caption is included in the file and not in the manuscript, and that the style conforms to the rest of the article.

# 2 Supplementary Figures and Tables

For more information on Supplementary Material and for details on the different file types accepted, please see [here](https://www.frontiersin.org/guidelines/author-guidelines#supplementary-material).

## 2.1 Supplementary Figures

**Supplementary Figure 1. Multivariable-adjusted associations of hyperthyroidism treatment subgroups with regional BMD.**

This forest plot presents the results of multivariable linear regression analyses comparing four mutually exclusive hyperthyroidism treatment subgroups (untreated [red], medication only [blue], surgery only [green], and medication plus surgery [gray]) against the healthy control group, with the healthy control group serving as the reference baseline (represented by the vertical dashed null line at 0). All point estimates are presented as adjusted regression coefficients (β) with their corresponding 95%CI across distinct regional skeletal sites. The models were fully and robustly adjusted using a stratified multiple imputation by chained equations (MICE, M = 20) protocol, controlling for a comprehensive clinical covariate matrix including age, sex, BMI, smoking status, alcohol consumption, physical activity index (IPAQ), vitamin D supplementation, and calcium supplementation.

**Supplementary Figure 2. Detailed cascade of hyperthyroidism-induced osteoporosis in GD.**

The diagram delineates the pathological sequence: TRAb-mediated thyroid overactivity → elevated T3/T4 → increased bone resorption and decreased formation → hypercalcemia → suppressed PTH → reduced renal and intestinal calcium reabsorption → negative calcium balance → net bone loss and decreased BMD.

**Supplementary Figure 3. **Dominant GD pathway versus vitamin D pathway.****

**(A) Vitamin D pathway:** Supplementation enhances intestinal calcium absorption and bone mineralization but fails to significantly improve BMD in GD patients.
**(B) GD-driven pathway:** Excessive T3/T4 potently stimulates osteoclastic bone resorption, leading to hypercalcemia, suppressed PTH, and marked BMD loss. Restoration of euthyroidism is essential for skeletal protection.

**Supplementary Figure 4. **Pathogenesis of bone loss in GD (pre- and post-treatment).****

**(A) Thyrotoxic phase:** TRAb-driven T3/T4 excess activates osteoclasts and inhibits osteoblasts, resulting in uncoupled bone remodeling, mineral dysregulation, and reduced BMD.
**(B) Euthyroid recovery phase:** Antithyroid therapy restores normal T3/T4 levels, rebalancing bone remodeling and mineral homeostasis, thereby facilitating BMD recovery.

**Supplementary Figure 5**. Proposed protective role of mechanical loading (body weight) in GD.****

Higher BMI may attenuate bone loss through mechanical stimulation of osteoblast activity and bone formation, partially counteracting the resorptive effects of thyroid hormone excess, highlighting the relevance of weight management in comprehensive GD care.

## 2.2 Supplementary Tables

**Supplementary Table 1. Quality Assessment of included Cross‑sectional Studies using the AHRQ 11‑item checklist**

| **Study** | **Time** | **Data source specified** | **Inclusion/exclusion listed** | **Time period given** | **Subjects consecutive** | **Subjective factors masked** | **Quality assurance described** | **Exclusion reasons explained** | **Confounding controlled** | **Missing data handled** | **Response rate/completeness** | **Follow-up addressed** | **Total** | **Quality** |
| --- | --- | --- | --- | --- | --- | --- | --- | --- | --- | --- | --- | --- | --- | --- |
| Boro (12) | 2023 | Yes | Yes | No | Unclear | Yes | Yes | Yes | Yes | Unclear | Yes | Yes | 8 | High |
| Lee,H.S (13) | 2021 | Yes | Yes | No | Unclear | Yes | Yes | Yes | Yes | Unclear | Unclear | Yes | 7 | High |
| Siderova (14) | 2018 | Yes | Yes | No | Yes | Yes | No | Yes | Yes | Unclear | Yes | Yes | 8 | High |
| Kuzma (15) | 2018 | Yes | Yes | No | Yes | No | No | Yes | Yes | Unclear | Yes | Yes | 8 | High |
| Biswas (16) | 2015 | Yes | Yes | Yes | Yes | No | Unclear | Yes | Yes | Unclear | No | No | 7 | High |
| Amashukeli (17) | 2013 | Yes | Yes | No | Unclear | Yes | Yes | Yes | Yes | Unclear | Unclear | Yes | 7 | High |
| Majima-2 (18) | 2006 | Yes | Yes | Yes | Unclear | No | Unclear | Yes | Yes | Unclear | No | Yes | 7 | High |
| Altun (19) | 2003 | Yes | Yes | No | Unclear | No | Unclear | Yes | Yes | Yes | No | Yes | 7 | High |
| Leary(I) (20) | 2001 | Yes | Yes | No | Unclear | No | No | Yes | Yes | Unclear | No | Yes | 6 | High |
| Leary(II) (20) | 2001 | Yes | Yes | No | Unclear | No | No | Yes | Yes | Unclear | No | Yes | 6 | High |
| Lee,M.S (I) (21) | 1990 | Yes | Yes | No | Unclear | Yes | Yes | Yes | Yes | Unclear | Unclear | Yes | 7 | High |
| Lee,M.S (II) (21) | 1990 | Yes | Yes | No | Unclear | Yes | Yes | Yes | Yes | Unclear | Unclear | Yes | 7 | High |
| Lee,M.S (III) (21) | 1990 | Yes | Yes | No | Unclear | Yes | Yes | Yes | Yes | Unclear | Unclear | Yes | 7 | High |
| Lee,M.S (IV) (21) | 1990 | Yes | Yes | No | Unclear | Yes | Yes | Yes | Yes | Unclear | Unclear | Yes | 7 | High |
| Ercolano(I) (22) | 2013 | Yes | Yes | Yes | Unclear | No | Unclear | Yes | Yes | Unclear | No | Yes | 7 | High |
| Ercolano(II) (22) | 2013 | Yes | Yes | Yes | Unclear | No | Unclear | Yes | Yes | Unclear | No | Yes | 7 | High |

AHRQ criteria for cross-sectional studies (11 items). The assessment domains include: (1) Data source specification; (2) Inclusion or exclusion criteria; (3) Time period of patient identification; (4) Consecutive enrollment of subjects; (5) Control of subjective bias (reverse-scored); (6) Quality assurance measures; (7) Reasons for exclusion; (8) Confounding control; (9) Missing data handling; (10) Response rate and data completeness; (11) Follow-up handling. Each item was scored as “Yes” (1 point), “No” (0 points), or “Unclear” (0 points). Total scores range from 0 to 11. Quality classification: 9–11= High quality; 5–8 = Moderate quality; 0–4 = Low quality.

**Supplementary Table 2. Quality Assessment of Included Cohort Studies Using the NOS**

| **Study** | **Time** | **Selection**  **(0-4)** | **Comparability**  **(0-2)** | **Outcome**  **(0-3)** | **Total** | **Quality** |
| --- | --- | --- | --- | --- | --- | --- |
| Zhang (23) | 2022 | 4 | 2 | 3 | 9 | High |
| Jyotsna (24) | 2012 | 4 | 2 | 2 | 8 | High |
| Majima-1 (II) (25) | 2006 | 4 | 1 | 3 | 8 | High |

Selection criteria (0–4): representativeness of the exposed cohort, selection of the non-exposed cohort, ascertainment of exposure, and outcome not present at baseline. Comparability (0–2): control for age/sex and additional confounders. Outcome (0–3): independent outcome assessment, adequate follow-up duration, and follow-up completeness. Total scores range from 0 to 9. Quality classification: 7–9 = High quality; 4–6 = Moderate quality; 0–3 = Low quality.

**Supplementary Table 3. Missing data proportions for covariates included in the multivariable models, shown separately for control and hyperthyroidism groups.**

| **Variable** | **Control_Group** | **Hyperthyroidism_Group** |
| --- | --- | --- |
| AGE | 0 (0%) | 0 (0%) |
| Alcohol | 281 (0.67%) | 4 (0.78%) |
| BMI | 1078 (2.58%) | 12 (2.34%) |
| Calcium | 0 (0%) | 0 (0%) |
| IPAQ | 6224 (14.87%) | 96 (18.75%) |
| Sex | 0 (0%) | 0 (0%) |
| Smoke | 281 (0.67%) | 4 (0.78%) |
| VitaminD | 0 (0%) | 0 (0%) |

Missing data proportions for most covariates were low, ranging from 0% to 0.78% in both groups, with the exception of IPAQ (14.87% in controls and 18.75% in cases), which is consistent with missing rates commonly observed in large-scale epidemiological cohorts. Missing data were handled using multiple imputation by chained equations (MICE) with 20 imputed datasets under the missing at random (MAR) assumption, with the imputation model including all 19 BMD outcome variables, the primary exposure (hyperthyroidism status), and all adjustment covariates.

**Supplementary Table 4. Stratified analysis of the association between hyperthyroidism and BMD by sex and menopausal status.**

| **BMD** | **Subgroup** | **Adjusted β** | **95% CI Lower** | **95% CI Upper** | **P** | **n(c)** | **n(Hyper)** |
| --- | --- | --- | --- | --- | --- | --- | --- |
| L1_L4 | All_Males | -6.00E-04 | -0.0364 | 0.0352 | 0.97299 | 21236 | 109 |
|  | Postmenopausal_Females | -7.00E-04 | -0.0181 | 0.0167 | 0.93643 | 19259 | 372 |
|  | Premenopausal_Females | -0.029 | -0.0908 | 0.0328 | 0.35385 | 1367 | 31 |
| FN_R | All_Males | 0.0018 | -0.0231 | 0.0267 | 0.88699 | 21236 | 109 |
|  | Postmenopausal_Females | -9.00E-04 | -0.0132 | 0.0114 | 0.88687 | 19261 | 372 |
|  | Premenopausal_Females | -0.0356 | -0.0822 | 0.0111 | 0.13471 | 1365 | 31 |
| FS_R | All_Males | -0.0079 | -0.0392 | 0.0235 | 0.62313 | 21236 | 109 |
|  | Postmenopausal_Females | -0.0113 | -0.0269 | 0.0043 | 0.15652 | 19254 | 373 |
|  | Premenopausal_Females | -0.0607 | -0.1189 | -0.0025 | 0.04081 | 1372 | 30 |
| TH_R | All_Males | -0.007 | -0.0328 | 0.0188 | 0.59568 | 21236 | 109 |
|  | Postmenopausal_Females | -0.0053 | -0.0178 | 0.0071 | 0.40119 | 19256 | 372 |
|  | Premenopausal_Females | -0.0448 | -0.0912 | 0.0017 | 0.05903 | 1370 | 31 |
| Troch_R | All_Males | -0.0062 | -0.0319 | 0.0195 | 0.63467 | 21236 | 109 |
|  | Postmenopausal_Females | -0.0011 | -0.0127 | 0.0105 | 0.8507 | 19257 | 372 |
|  | Premenopausal_Females | -0.0227 | -0.0664 | 0.0211 | 0.30837 | 1369 | 31 |
| Wards_R | All_Males | 0.0111 | -0.0159 | 0.0381 | 0.42067 | 21236 | 109 |
|  | Postmenopausal_Females | -0.0039 | -0.0171 | 0.0093 | 0.56408 | 19261 | 372 |
|  | Premenopausal_Females | -0.0353 | -0.0883 | 0.0176 | 0.19043 | 1365 | 31 |
| Arms | All_Males | -0.0113 | -0.0302 | 0.0076 | 0.23978 | 21236 | 109 |
|  | Postmenopausal_Females | -8.00E-04 | -0.0098 | 0.0083 | 0.86947 | 19253 | 372 |
|  | Premenopausal_Females | -0.0217 | -0.0529 | 0.0095 | 0.17204 | 1373 | 31 |
| Head | All_Males | 0.0017 | -0.0538 | 0.0571 | 0.95232 | 21236 | 109 |
|  | Postmenopausal_Females | 0.0132 | -0.0198 | 0.0462 | 0.43368 | 19260 | 372 |
|  | Premenopausal_Females | -0.0276 | -0.1633 | 0.1081 | 0.68068 | 1366 | 31 |
| Legs | All_Males | -0.008 | -0.0329 | 0.0168 | 0.52519 | 21236 | 109 |
|  | Postmenopausal_Females | -0.0049 | -0.0163 | 0.0065 | 0.39917 | 19262 | 372 |
|  | Premenopausal_Females | -0.028 | -0.0683 | 0.0122 | 0.17154 | 1364 | 31 |
| Pelvis | All_Males | -0.0137 | -0.0405 | 0.013 | 0.31328 | 21236 | 109 |
|  | Postmenopausal_Females | -8.00E-04 | -0.0131 | 0.0114 | 0.8938 | 19252 | 372 |
|  | Premenopausal_Females | -0.0378 | -0.0877 | 0.0122 | 0.13687 | 1374 | 31 |
| Ribs | All_Males | -0.0168 | -0.0345 | 0.001 | 0.06498 | 21236 | 109 |
|  | Postmenopausal_Females | -0.0076 | -0.0168 | 0.0016 | 0.10403 | 19254 | 372 |
|  | Premenopausal_Females | -0.0448 | -0.0868 | -0.0029 | 0.03656 | 1372 | 31 |
| Spine | All_Males | -0.0077 | -0.0359 | 0.0205 | 0.59311 | 21236 | 109 |
|  | Postmenopausal_Females | 0.0025 | -0.0108 | 0.0158 | 0.71059 | 19250 | 372 |
|  | Premenopausal_Females | -0.0208 | -0.0739 | 0.0324 | 0.43786 | 1376 | 31 |
| Total | All_Males | -0.0093 | -0.0309 | 0.0122 | 0.39578 | 21236 | 109 |
|  | Postmenopausal_Females | -0.0017 | -0.0129 | 0.0095 | 0.76735 | 19256 | 372 |
|  | Premenopausal_Females | -0.0318 | -0.0714 | 0.0078 | 0.11443 | 1370 | 31 |
| Trunk | All_Males | -0.0116 | -0.0336 | 0.0104 | 0.30227 | 21236 | 109 |
|  | Postmenopausal_Females | -0.0017 | -0.0122 | 0.0087 | 0.74474 | 19251 | 372 |
|  | Premenopausal_Females | -0.0335 | -0.074 | 0.007 | 0.10457 | 1375 | 31 |
| FS_L | All_Males | -0.0117 | -0.043 | 0.0196 | 0.46479 | 21236 | 109 |
|  | Postmenopausal_Females | -0.0102 | -0.0256 | 0.0052 | 0.19329 | 19254 | 373 |
|  | Premenopausal_Females | -0.065 | -0.1309 | 8.00E-04 | 0.05283 | 1372 | 30 |
| TH_L | All_Males | -0.0106 | -0.0365 | 0.0153 | 0.42187 | 21236 | 109 |
|  | Postmenopausal_Females | -0.0043 | -0.0164 | 0.0079 | 0.49311 | 19254 | 373 |
|  | Premenopausal_Females | -0.0452 | -0.095 | 0.0045 | 0.07468 | 1372 | 30 |
| Troch_L | All_Males | -0.0093 | -0.0351 | 0.0165 | 0.48145 | 21236 | 109 |
|  | Postmenopausal_Females | -4.00E-04 | -0.0121 | 0.0113 | 0.95146 | 19263 | 372 |
|  | Premenopausal_Females | -0.0237 | -0.0705 | 0.0231 | 0.31906 | 1363 | 31 |
| Wards_L | All_Males | 0.0012 | -0.0255 | 0.0278 | 0.93236 | 21236 | 109 |
|  | Postmenopausal_Females | -0.0027 | -0.0159 | 0.0105 | 0.68859 | 19266 | 372 |
|  | Premenopausal_Females | -0.0431 | -0.0964 | 0.0102 | 0.11274 | 1360 | 31 |
| FN_L | All_Males | -0.007 | -0.0318 | 0.0178 | 0.57896 | 21236 | 109 |
|  | Postmenopausal_Females | 0 | -0.0124 | 0.0123 | 0.9979 | 19266 | 373 |
|  | Premenopausal_Females | -0.0459 | -0.1023 | 0.0105 | 0.10838 | 1360 | 30 |

All values represent the multivariable-adjusted β and their 95% CI obtained from linear regression models comparing individuals with hyperthyroidism versus healthy reference controls within each respective demographic stratum. To control for potential confounding and minimize residual bias while preserving strata-specific variance structures, a stratified multiple imputation by chained equations (MICE, M = 20) protocol was deployed independently within the male and female subgroups under the missing at random (MAR) assumption. Model specifications are identical across all strata, with the regressions fully adjusted for age, BMI, smoking status, alcohol consumption, IPAQ, vitamin D supplementation, and calcium supplementation. Menopausal status was further included in female-specific stratifications to separate premenopausal and postmenopausal pathways.

**Table 5. Change-in-estimate analysis quantifying the contribution of each covariate to the attenuation of the hyperthyroidism-BMD association.**

| **BMD** | **Variable** | **Change_Percentage** | **Standardized_β_Impact** |
| --- | --- | --- | --- |
| Arms | Sex | -89.55% | 0.7017 |
|  | BMI | 1.13% | 0.2212 |
|  | AGE | -2.27% | 0.1931 |
|  | IPAQ | 0.19% | 0.0555 |
|  | Alcohol | -5.51% | 0.034 |
|  | Calcium | -5.50% | 0.0183 |
|  | VitaminD | -3.85% | 0.0174 |
|  | Smoke | 1.03% | 0.0037 |
| FN_L | Sex | -75.03% | 0.2996 |
|  | BMI | 1.77% | 0.2606 |
|  | AGE | -6.31% | 0.2167 |
|  | IPAQ | -0.27% | 0.0813 |
|  | Alcohol | -6.04% | 0.0606 |
|  | VitaminD | -5.22% | 0.0211 |
|  | Calcium | -7.63% | 0.0181 |
|  | Smoke | 0.80% | 0.009 |
| FN_R | Sex | -80.19% | 0.3116 |
|  | BMI | 2.02% | 0.2554 |
|  | AGE | -6.47% | 0.2162 |
|  | IPAQ | -0.04% | 0.0815 |
|  | Alcohol | -6.32% | 0.0581 |
|  | VitaminD | -5.64% | 0.0227 |
|  | Calcium | -8.05% | 0.0182 |
|  | Smoke | 0.75% | 0.0108 |
| FS_L | BMI | 1.91% | 0.3371 |
|  | Sex | -68.25% | 0.3132 |
|  | AGE | -4.60% | 0.1878 |
|  | IPAQ | 0.71% | 0.0706 |
|  | Alcohol | -3.92% | 0.0465 |
|  | VitaminD | -4.58% | 0.0211 |
|  | Calcium | -6.91% | 0.021 |
|  | Smoke | 0.62% | 0.0146 |
| FS_R | Sex | -69.75% | 0.3304 |
|  | BMI | 1.58% | 0.3191 |
|  | AGE | -4.65% | 0.1957 |
|  | IPAQ | 0.67% | 0.067 |
|  | Alcohol | -4.19% | 0.049 |
|  | VitaminD | -4.53% | 0.0203 |
|  | Calcium | -6.70% | 0.0199 |
|  | Smoke | 0.57% | 0.0171 |
| Head | BMI | 2.33% | 0.1478 |
|  | Sex | -115.58% | 0.1385 |
|  | AGE | -14.40% | 0.1344 |
|  | Calcium | -23.08% | 0.0266 |
|  | Smoke | -1.35% | 0.026 |
|  | VitaminD | -14.72% | 0.0231 |
|  | IPAQ | 7.07% | 0.008 |
|  | Alcohol | 0.04% | 0.0053 |
| L1_L4 | Sex | -96.78% | 0.3212 |
|  | BMI | 1.98% | 0.2676 |
|  | Alcohol | -8.04% | 0.0586 |
|  | IPAQ | 1.22% | 0.0431 |
|  | Calcium | -9.67% | 0.0295 |
|  | VitaminD | -6.62% | 0.0253 |
|  | AGE | 1.26% | 0.0127 |
|  | Smoke | 1.59% | 0.0036 |
| Legs | Sex | -86.31% | 0.6797 |
|  | BMI | 0.86% | 0.2207 |
|  | AGE | -2.01% | 0.1764 |
|  | IPAQ | -0.34% | 0.0803 |
|  | Alcohol | -6.06% | 0.0563 |
|  | Calcium | -5.57% | 0.0207 |
|  | VitaminD | -3.93% | 0.0198 |
|  | Smoke | 0.68% | 0.0138 |
| Pelvis | Sex | -79.39% | 0.4196 |
|  | BMI | 1.13% | 0.2404 |
|  | AGE | -3.86% | 0.1896 |
|  | IPAQ | -0.46% | 0.0829 |
|  | Alcohol | -7.17% | 0.0802 |
|  | VitaminD | -4.95% | 0.0242 |
|  | Calcium | -7.02% | 0.0227 |
|  | Smoke | 0.65% | 0.014 |
| Ribs | Sex | -81.61% | 0.5734 |
|  | BMI | 1.48% | 0.4389 |
|  | AGE | -2.08% | 0.1544 |
|  | Alcohol | -4.42% | 0.05 |
|  | IPAQ | 2.05% | 0.0208 |
|  | Calcium | -5.64% | 0.0197 |
|  | VitaminD | -3.83% | 0.0191 |
|  | Smoke | 1.09% | 0.0101 |
| Spine | Sex | -101.32% | 0.4906 |
|  | BMI | 1.34% | 0.3668 |
|  | IPAQ | 1.35% | 0.0548 |
|  | Alcohol | -6.03% | 0.0456 |
|  | VitaminD | -5.21% | 0.0225 |
|  | AGE | 0.28% | 0.0223 |
|  | Calcium | -7.34% | 0.0223 |
|  | Smoke | 1.53% | 0.0044 |
| TH_L | Sex | -79.15% | 0.4013 |
|  | BMI | 1.58% | 0.336 |
|  | AGE | -3.61% | 0.1729 |
|  | IPAQ | 0.66% | 0.0746 |
|  | Alcohol | -5.73% | 0.0645 |
|  | Calcium | -6.64% | 0.0197 |
|  | VitaminD | -4.52% | 0.0195 |
|  | Smoke | 0.82% | 0.0143 |
| TH_R | Sex | -78.45% | 0.4041 |
|  | BMI | 1.76% | 0.3213 |
|  | AGE | -3.81% | 0.1827 |
|  | Alcohol | -5.83% | 0.066 |
|  | IPAQ | 0.59% | 0.0642 |
|  | VitaminD | -4.52% | 0.0203 |
|  | Calcium | -6.60% | 0.0199 |
|  | Smoke | 0.74% | 0.0178 |
| Total | Sex | -85.63% | 0.5788 |
|  | BMI | 1% | 0.2636 |
|  | AGE | -2.80% | 0.1884 |
|  | IPAQ | 0.30% | 0.0654 |
|  | Alcohol | -5.61% | 0.0517 |
|  | Calcium | -6.53% | 0.025 |
|  | VitaminD | -4.55% | 0.0244 |
|  | Smoke | 0.68% | 0.014 |
| Troch_L | Sex | -92.62% | 0.5267 |
|  | BMI | 1.25% | 0.3093 |
|  | AGE | -1.09% | 0.0908 |
|  | Alcohol | -7.61% | 0.0813 |
|  | IPAQ | 0.45% | 0.0591 |
|  | Calcium | -5.92% | 0.0164 |
|  | VitaminD | -4.14% | 0.0151 |
|  | Smoke | 1.11% | 0.0139 |
| Troch_R | Sex | -91.48% | 0.5192 |
|  | BMI | 1.27% | 0.306 |
|  | AGE | -1.36% | 0.102 |
|  | Alcohol | -7.84% | 0.0847 |
|  | IPAQ | 0.46% | 0.0624 |
|  | Calcium | -5.88% | 0.0157 |
|  | Smoke | 1.04% | 0.0154 |
|  | VitaminD | -4.13% | 0.0152 |
| Trunk | Sex | -86.36% | 0.5156 |
|  | BMI | 1.37% | 0.3512 |
|  | AGE | -2.03% | 0.1329 |
|  | Alcohol | -5.98% | 0.0651 |
|  | IPAQ | 0.90% | 0.0593 |
|  | VitaminD | -4.64% | 0.0241 |
|  | Calcium | -6.59% | 0.0226 |
|  | Smoke | 1.05% | 0.0107 |
| Wards_L | AGE | -9.17% | 0.256 |
|  | Sex | -67.76% | 0.2391 |
|  | BMI | 1.88% | 0.2085 |
|  | IPAQ | 0.27% | 0.062 |
|  | Alcohol | -4.38% | 0.0437 |
|  | VitaminD | -5.96% | 0.0236 |
|  | Smoke | -0.10% | 0.02 |
|  | Calcium | -8.66% | 0.0191 |
| Wards_R | AGE | -10.22% | 0.263 |
|  | Sex | -71.23% | 0.2342 |
|  | BMI | 1.83% | 0.1957 |
|  | IPAQ | 0.48% | 0.0511 |
|  | Alcohol | -4.52% | 0.0417 |
|  | VitaminD | -6.58% | 0.0249 |
|  | Smoke | -0.26% | 0.0198 |
|  | Calcium | -9.43% | 0.0192 |

The “Change_Percentage” (Δβ%) denotes the confounding intensity of each individual covariate on the primary exposure effect. It was calculated by sequentially adding each covariate to the null model containing only the exposure (hyperthyroidism status) and tracking the relative change in the regression coefficient (β) for hyperthyroidism compared with the unadjusted baseline model: Δβ% = [(βcovariate - βnull) / βnull] × 100%. An absolute change of ≥ 10% is conventionally considered an indicator of a meaningful confounding effect. The “Standardized_β_Impact” represents the independent association of each covariate with BMD outcomes. It was extracted from the fully adjusted multivariable linear regression model after Z-score transformation of continuous variables (age and BMI); for categorical covariates (smoking, alcohol, IPAQ, vitamin D supplementation, and calcium supplementation), the maximum absolute standardized coefficient among the internal categories was reported to ensure comparability across covariates. Abbreviations: FN, femoral neck; FS, femoral shaft; TH, total hip; Troch, trochanter; L, left; R, right.
